# Perturbation of the yeast N-acetyltransferase NatB induces elevation of protein phosphorylation levels

**DOI:** 10.1186/1471-2164-11-685

**Published:** 2010-12-02

**Authors:** Andreas O Helbig, Sara Rosati, Pim WWM Pijnappel, Bas van Breukelen, Marc HTH Timmers, Shabaz Mohammed, Monique Slijper, Albert JR Heck

**Affiliations:** 1 Biomolecular Mass Spectrometry and Proteomics Group, Utrecht Institute for Pharmaceutical Sciences and Bijvoet Center for Biomolecular Research, Utrecht University, Padualaan 8, Utrecht, 3584 CH, The Netherlands; 2 Netherlands Proteomics Centre, Padualaan 8, Utrecht, 3584 CH, The Netherlands; 3 University Medical Center Utrecht, Universiteitsweg 100, Utrecht, 3584 CG, The Netherlands; 4 Center for Biomedical Genetics, MCU, Stratenum 3.223, Universiteitsweg 100, Utrecht, 3584 CG, The Netherlands; 5 Netherlands Bioinformatics Centre, Geert Grooteplein 28, Nijmgen, 6525 GA, The Netherlands

## Abstract

**Background:**

The addition of an acetyl group to protein N-termini is a widespread co-translational modification. NatB is one of the main N-acetyltransferases that targets a subset of proteins possessing an N-terminal methionine, but so far only a handful of substrates have been reported. Using a yeast *nat3Δ *strain, deficient for the catalytic subunit of NatB, we employed a quantitative proteomics strategy to identify NatB substrates and to characterize downstream effects in *nat3Δ*.

**Results:**

Comparing by proteomics WT and *nat3Δ *strains, using metabolic ^15^N isotope labeling, we confidently identified 59 NatB substrates, out of a total of 756 detected acetylated protein N-termini. We acquired in-depth proteome wide measurements of expression levels of about 2580 proteins. Most remarkably, NatB deletion led to a very significant change in protein phosphorylation.

**Conclusions:**

Protein expression levels change only marginally in between WT and *nat3Δ*. A comparison of the detected NatB substrates with their orthologous revealed remarkably little conservation throughout the phylogenetic tree. We further present evidence of post-translational N-acetylation on protein variants at non-annotated N-termini. Moreover, analysis of downstream effects in *nat3Δ *revealed elevated protein phosphorylation levels whereby the kinase Snf1p is likely a key element in this process.

## Background

Post translational modifications of proteins are important events that influence protein function, interaction and localization [1], making those key elements in cellular processes and systemic reactions of organisms. The transfer of an acetyl group from acetyl-coenzyme A to the α-amino group of an N-terminal amino acid residue is a very common modification that occurs on a large part of the proteome (i.e. about 50% of yeast proteins and up to 90% in mammals) [2], [3]. This modification can be carried out by one of five protein complexes (NatA, NatB, NatC, NatD and NatE), whereby each consists of a catalytic and a varying number of auxiliary subunits [4]. The function of these complexes seems to be highly conserved across species [5]. For yeast NatB, which will be the target of this study, the complex consists of Nat3p (catalytic subunit) and Mdm20p (auxiliary subunit) [6]. N-acetyltransferase complexes act upon the N-terminus of polypeptide chains at the ribosome during their synthesis [7]. They work in conjunction with methionine amino peptidases that can cleave the initial methionine dependent on the penultimate amino acid residue [8], [9]. The substrate recognition of the different N-acetyl transferases is primarily dependent on the N-terminal amino acid sequence of target proteins [10]. However, other (co-)factors may play a role. For instance, the Huntingtin (Htt) interacting protein HYPK, which associates with NatA in human cells, is required for N-acetylation of certain NatA targets [11].

The best characterized N-acetyltransferases (NATs) are NatA, NatB and NatC. NatA acetylates the largest set of proteins, which have had their initial methionine removed and possess predominantly a serine, alanine, threonine, valine or glycine at their N-terminus [10]. The substrates of NatB and NatC still contain the N-terminal methionine whereby the specificity of these N-acetyltransferases is directly dependent on the penultimate amino acid. NatB targets proteins that display a glutamic acid, aspartic acid or glutamine in the penultimate position while NatC seems to prefer isoleucine, leucine, tryptophan and phenylalanine at the penultimate position [4].

In a number of studies protein N-acetylation in yeast has been charted [5], [12], [10], [13]. However, the overall coverage and characterization of the yeast N-acetylated proteome is still far from complete. For instance, for the N-terminal acetyltransferase complex NatB, subject of this study, only 14 substrates have been experimentally verified so far. Mutants deficient for NatA, NatB or NatC are viable but they generally display defects in aspects such as growth, temperature sensitivity and sporulation. Further, Polevoda *et al*. showed that the *nat3Δ *displays temperature sensitivity and reduced growth on glycerol and NaCl containing media [14]. Despite targeting a significantly smaller subset of proteins, the phenotype of a NatB (*nat3Δ*) knockout is much more apparent than the phenotype for a NatA (*nat1Δ*) deficient strain. In the case of NatB deficiency, the effects cover decreased resistance to chemicals, abnormal budding, increased cell size and a decreased growth rate [13]. Caesar *et al*. [13] proposed that putative NatB targets are preferentially involved in cell cycle progression and maintenance of the nucleus. It has been shown, for instance, that the N-acetylation of the NatB target tropomyosin is necessary for its association with actin [15]. Here the N-acetylation is thought to induce a conformational change that stabilizes coiled-coil structures involved in tropomyosin-actin polymerization. Restoring the actin filaments did not suppress the NatB phenotype, indicating a complex interplay of multiple NatB related effects on different proteins. Another study demonstrated that N-acetylation of the CPY inhibitor Tfs1 is necessary for its inhibitory function [16]. Most recently, it was suggested that protein N-acetylation can act as a degradation signal recognized by the Doa10p ubiquitin ligase [17]. This implies that protein N-acetylation can also be involved in protein stability. All this recent work indicates that the complex and diverse role of protein N-terminal acetylation is slowly more and more revealed.

Traditionally, N-acetylated proteins were identified by their change in electrophoretic mobility, for instance on 2 D gels. New experimental strategies like the diagonal chromatography COFRADIC approach now allow for the enrichment and quantitative characterization of protein N-acetylation at a much higher through-put [18], [19]. COFRADIC sorting of N-acetylated peptides enabled the large-scale charting of protein N-acetylation in human cell lines[20], *Drosophila melanogaster *[21] and even the prokaryotes *Halobacterium salinarum *and *Natronomonas pharaonis *[22]. Another technique amendable for the targeted analysis of protein N-termini involves the coupling of free N-terminal amine groups to CNBr activated sepharose [23] or dendritic polyglycerol aldehyde polymers [24]. This allows the subsequent removal of all "normal" peptides enriching the N-terminally modified peptide subset. Recently, we introduced a straightforward methodology, based solely on strong cation exchange (SCX) that is able to achieve near baseline separation of N-acetylated [25], phosphorylated and unmodified peptide populations [26], [27], and applied this technique to characterize for instance the N-acetylated proteome of HEK293 cells [9].

Here, we extend the use of this technology, in conjunction with metabolic ^15^N stable isotope labeling [28], to experimentally identify NatB substrates and to investigate the effects of NatB mediated protein N-acetylation on the *S. cerevisiae *proteome. Employing a comprehensive mass spectrometry based strategy that utilizes the complementarity between trypsin and Lys-N proteases we map differential protein abundances, protein phosphorylation and N-terminal acetylation in a WT and *nat3Δ *yeast strain, in an effort to investigate in more depth the role of protein N-terminal acetylation.

## Methods

### Cell culturing

*Saccharomyces cerevisiae *strains were purchased from Euroscarf (University of Frankfurt, Germany). Yeast wildtype (BY4742, MATα, his3Δ1, leu2Δ0, met15Δ0, ura3Δ0) and NAA20 (Nat3) knockout (BY4742, MATα, his3Δ1, leu2Δ0, met15Δ0, ura3Δ0, YPR131C::kanMX4) strains were cultured on YNB medium (medium base 1.72 g/l), which was supplied with a 20 amino acid mix (1.4 g/l) and glucose (20 g/l). Ammoniumsulphate (5 g/l) was used as a nitrogen source. Both yeast strains were grown on "regular" and "heavy" medium, containing ^15^N labeled ammoniumsulphate and ^15^N labeled amino acid supplements (Sigma Isotech). After growth on selective plates, both strains were cultured in shake flasks to a similar optical density in the exponential growth phase (OD between 1 and 2). Subsequently cells were harvested, washed twice with water and subjected to lyophilization.

### Sample preparation

Wildtype and mutant lyophilized material (a biological replicate experiment was conducted with reversed isotopic labels) was mixed 1:1 based on dry weight. A total of 50 mg mixed biomass was resuspended in 200 μl of lysis buffer containing 4% SDS, 25% glycerol, 138 mM Tris-HCL pH 6.8 and 200 mM DTT. After the addition of glass beads, the solution was kept on ice and subsequently vortexed 5 times for 2 min to solubilize proteins. The supernatant was then centrifuged at 1000 g for 5 min. Solubilized proteins were cast in a polyacrylamide gel matrix without electrophoresis. The gel was cut into small pieces, fixed (30% methanol, 20% acetic acid) and washed extensively with 50 mM ammonium bicarbonate. Reduction and alkylation was carried out as previously described for in gel digestion using Lys-N and trypsin[29], [30]. After overnight digestion, peptides were extracted from the gel by the addition of 100% acetonitrile, which was removed from the sample by vacuum evaporation prior to strong cation exchange chromatography of peptides.

### Strong cation exchange

Approximately 1.5 mg of peptide material was loaded onto 2 C18 Opti-Lynx cartridges, using an Agilent 1100 HPLC system, at a flow rate of 200 μl/min in 0.05% FA. Elution from the trapping cartridges was achieved using 80% acetonitrile/0.05% FA and loaded onto a PolySULFOETHYL A column 200 × 2.1 mm (PolyLC inc.) for 10 minutes at the same flow rate. The different peptide populations were separated using a non-linear 65 minute gradient at 200 μl/minute of solvent A (5 mM KH_2_PO_4_, 30% Acetonitrile, 350 mM KCl, 0.05% FA) and solvent B (5 mM KH_2_PO_4_, 30% Acetonitrile, 0.05% FA). From 0 to 10 minutes isocratic flow of 100% solvent A was performed, from 10 to 15 minutes a linear gradient up to 26% solvent B, from 15 to 40 minutes a linear gradient to 35% solvent B from 40 to 45 minutes a linear gradient to 60% solvent reaching 100% solvent B at 49 minutes. The column was then washed for 6 minutes with 100% solvent B and finally equilibrated with 100% solvent A for 9 minutes. Fractions were collected at one minute intervals for 40 minutes, dried and re-suspended in 40 μl 10% formic acid. 20 μl of each fraction (5 μl for the major +2 fractions) were used for further analysis.

### Mass spectrometry

The LC-MS/MS analysis was performed using a nano LC-LTQ-Orbitrap (Thermo, San Jose, CA) and an Agilent 1200 series LC system equipped with a 20 mm Aqua C18 trapping column (packed in-house, i.d., 100 μm; resin, 5 μm) and a 400 mm ReproSil-Pur C18-AQ analytical column (packed in-house, i.d., 50 μm; resin, 3 μm). Trapping was performed at 5 μL/min for 10 min in solvent A (0.1 M acetic acid in water), and elution was achieved with a linear gradient of 10-35% B (0.1 M acetic acid in 80/20 acetonitrile/water) for 90 minutes with a total analysis time of 120 minutes. The flow rate was passively split to 100 nL/min during the gradient analysis. Nanospray was achieved using a distally coated fused silica emitter (New Objective, Cambridge, MA) (o.d., 360 μm; i.d., 20 μm, tip i.d. 10 μm) biased to 1.7 kV. A 33MΩ resistor was introduced between the high voltage supply and the electrospray needle to reduce the ion current. The LTQ-Orbitrap mass spectrometer was operated in data-dependent mode, automatically switching between MS and MS/MS. Full scan MS spectra (300-1500 m/z) were acquired with a resolution of 60,000 at 400 m/z and accumulation to a target value of 500,000. The five most intense peaks above a threshold of 500 were selected for collision induced dissociation in the linear ion trap at normalized collision energy of 35 after accumulation to a target value of 30,000.

### Data processing

As described in reference 9, all MS and MS/MS spectra were searched using the MASCOT search engine (Matrix Science, London, UK, v.2.2.04) against the yeast SGD database (http://www.yeastgenome.org, 2009) containing 5779 entries. ^15^N metabolic labeling was selected as quantitation mode in MASCOT. Trypsin and Lys-N were chosen appropriately as proteolytic enzyme allowing one missed cleavage. N-terminal acetylation was chosen as a variable modification. Additionally, the data was searched using semi-trypsin or semi-Lys-N as enzyme and N-terminal acetylation as variable modification. Calculation of false-discovery-rates (FDR) was performed according to [27]. For phosphopeptide identification, the data was searched using trypsin and Lys-N as enzyme and phosphorylation on serine, threonine and tyrosine residues was chosen as variable modifications. A PTM score was assigned for each phosphopeptide above with MSQUANT version 1.5a61 [31]. Relative quantification of ^14^N and ^15^N peptide MS^1 ^intensities was performed using MSQUANT version 1.5a61. Ratios were subsequently ^2^log transformed and averaged between the two experiments. Only regular and N-acetylated peptides showing a MASCOT ion score above 30 were kept in the datasets to ensure a FDR below 1%. For phosphopeptides a minimum MASCOT score of 25 was chosen. To evaluate reproducibility, a 95% confidence interval was calculated for peptides quantified in both biological replicates [32]. Network analysis was performed using STRING v8.2 on high stringency setting [33] and the extraction of main protein interaction clusters was performed using MCODE v1.2 [34] and Cytoscape v2.6.3 [35]. Prediction of kinases was performed using NetworKIN v2.0 [36] and protein localization information was retrieved from the SGD database. Amino acid frequency analysis of N-terminal peptide sequences were calculated using Weblogo http://weblogo.berkeley.edu. Corrected p-values for overrepresented predicted kinases were calculated using the Pearson's chi-square test.

### N-terminal amino acid conservation

To determine the level of site conservation of the NatB substrate recognition motifs, MD, ME and MN, the orthologous sequences of 59 NatB substrates were retrieved from EGGNOG v2.0 [37]. Only eukaryotic species (52 in total) were included for analysis. Per species it was counted which percentage of the total sequences started with MD, ME or MN to obtain the level of NatB substrate conservation. Additionally this was also determined for every NatB substrate across species to determine if certain proteins are more evolutionary conserved. The top five proteins that showed the highest conservation were separately analyzed as above to determine if these proteins show higher cross-species conservation.

All mass spectrometry data was loaded into Scaffold v.2 (Proteome Software, Portland, USA) and the data associated with this manuscript may be downloaded from http://ProteomeCommons.org Tranche using the following hash:

f9XjmbCVZwessddnJXDrKqDBiGTCEoLvFvr2v0zKnl5+TpH29Un/pvJQscS4JCLh4IJEyr6f1yz/32CpHeORp2UTTgMAAAAAAAAKXw==

## Results

### Yeast N-acetylome and primary nat3Δ effect

To investigate the primary and secondary effects of the loss of NatB mediated protein N-acetylation, we conducted a systemic quantitative proteome analysis using differential ^15^N labeling of WT and *nat3Δ *strains. Trypsin and Lys-N digestions were performed to increase proteome coverage and a refined strong cation exchange chromatographic separation was employed to separate and enrich N-acetylated, phosphorylated and unmodified peptides. Cumulatively, we identified 21375 unique peptides (17261 unmodified, 989 N-acetylated and 3125 phosphorylated). These corresponded to 2747 proteins and 756 unique N-acetylated protein N-termini (Additional file 1). Up to now 363 protein N-termini have been reported to be fully or partially acetylated in yeast (compiled by Arnesen *et al*. [20]). In our data we could confirm 165 of these termini and additionally, we expanded the known N-acetylated yeast proteome by additional 591 N-termini providing the most comprehensive catalogue of yeast protein N-terminal acetylations to date.

**Figure 1:**
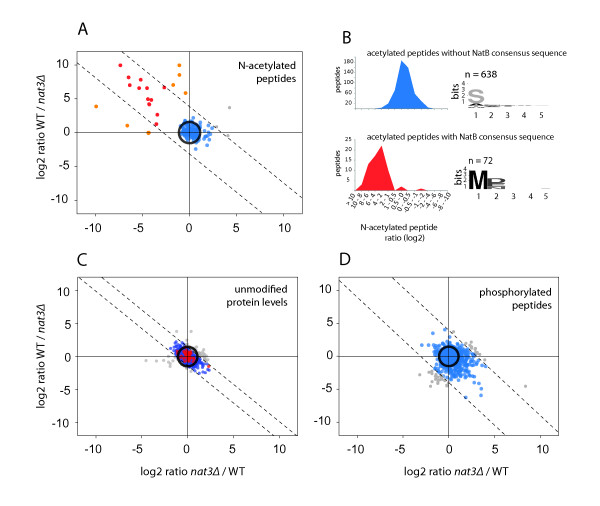
**Differential quantitation of 2560 proteins in the yeast WT/*nat3Δ *proteome enables identification of NatB substrates and reveals overall increased phosphorylation levels**. **Panel (A)**, **(C) **and **(D) **display peptide and protein ^15^N/^14^N ratios (^2^log transformed) determined in both biological replicates. Data of the two biological replicates are plotted *versus *each other. In experiment 1 the ΔNat3 strain was labeled with ^14^N while WT incorporated the heavy ^15^N label. In experiment 2 the isotope labels were reversed. The dashed lines represent a 95% confidence interval indicating high reproducibility of ratio data between biological replicates [32]. The circles indicate the chosen arbitrary thresholds for diminished or elevated protein levels, which were set at a three-fold change. **Panel (A) **displays ^15^N/^14^N ratio data of N-acetylated peptides, red colored spots mark N-acetylated peptides displaying the NatB target sequence while the lighter red indicates peptides located outside the 95% confidence. **Panel (B) **displays ^15^N/^14^N ratio histograms. The upper histogram shows ratios for all detected N-acetylated peptides not containing the expected NatB substrate sequence. The lower plot illustrates the ratio distribution of N-acetylated peptides containing the expected NatB substrate sequence, namely a methionine at the ultimate and an aspartic acid, glutamic acid or an asparagine in the penultimate position. Individual ratios from the biological replicates were averaged. The insets show frequency plots of the amino acids in the first 5 positions of the N-terminus generated by Weblogo. **Panel (C) **displays protein ratios as determined from unmodified peptides, with in red again the observed NatB substrate proteins. **(D) **displays phosphopeptide ratios, irrespective of being NatB substrate or not.

Using ^14^N/^15^N peptide ion intensities from WT and *nat3Δ *we obtained quantitative information on 2663 unmodified proteins (Additional file 2), 564 acetylated protein N-termini and 2309 phosphorylated sites (Additional file 3) (Figure 1A, C, D). Quantification data from the biological replicates showed very consistent and reproducible results since only a low number of outliers (4-7%) were observed outside a 95% confidence interval. ^15^N/^14^N ratios revealed that N-acetylated peptides with a NatB specific N-terminal sequence (ac-MDX, ac-MEX, ac-MNX) showed drastic down-regulation in the *nat3Δ *strain, verified in the biological replicate (Figure 1A). In total, 69 N-terminal peptides corresponding to 59 unique proteins (listed in Table 1) were detected with very significant decreased levels in the *nat3Δ *strain (Additional file 4) (Figure 1B). They all possessed the NatB specific N-terminal sequence. Since only 14 NatB substrates had been reported up to date, this is quite an expansion of experimentally verified NatB substrates. Of these 14 we could find 8 back in our study (Additional file 4). Strikingly, at the protein expression level, the detected NatB substrates were for the most part unchanged in the *nat3Δ *(Figure 1C) suggesting that the expression and/or degradation of these proteins is not significantly affected by N-acetylation. This indicates that NatB mediated N-acetylation does not act as a general degradon signal as suggested by Hwang et al. [17]. An initial network and clustering analysis of these 59 NatB substrates indicated that they can be found indiscriminately in different cellular localizations, e.g. the nucleus (e.g. Nsp1p, Nup84p or Rnr4p), the endoplasmatic reticulum/Golgi (e.g. Sec23p, Ypt1p or Bos1p) and the cytoplasm (e.g. Glc7p, Bud27p or Rpt3p).

**Table 1:** Detected NatB substrates

accession	name	score	sequence	start	average ratio
YLL026W	HSP104	65	MNDQT	1	**-8.7**
YPL111W	CAR1	66	METGP	1	**-7.3**
YDL029W	ARP2	54	MDPHN	1	**-6.5**
YGR078C	PAC10	47	MDTLF	1	**-6.0**
YJL136C	RPS21B	88	MENDK	1	**-5.6**
YER133W	GLC7	74	MDSQP	1	**-4.7**
YGR180C	RNR4	44	MEAHN	1	**-4.5**
YPR181C	SEC23	51	MDFET	1	**-4.3**
YLR078C	BOS1	51	MNALY	1	**-3.0**
YOR045W	TOM6	117	MDGMF	1	**-2.4**
YOR027W	STI1	42	MDDIN	198	**-0.7**
YER055C	HIS1	55	MDLVN	1	**-6.9**
YCL001W	RER1	97	MDYDS	1	**-5.5**
YDL100C	GET3	39	MDLTV	1	**-4.8**
YKR057W	RPS21A	96	MENDK	1	**-4.4**
YDR394W	RPT3	43	MEELG	1	**-3.8**
YBL082C	ALG3	67	MEGEQ	1	**-3.1**
YDR470C	UGO1	89	MNNNN	1	**-2.7**
YBR143C	SUP45	40	MDNEV	1	**-9.6**
YNL189W	SRP1	67	MDNGT	1	**-8.0**
YLR264W	RPS28B	32	MDSKT	1	**-8.0**
YLR438C-A	LSM3	40	METPL	1	**-7.5**
YHR028C	DAP2	69	MEGGE	1	**-7.4**
YLR118C		48	MNGLR	1	**-7.1**
YMR074C		60	MDPEL	1	**-6.9**
YGR275W	RTT102	30	MDPQT	1	**-6.3**
YFL038C	YPT1	40	MNSEY	1	**-6.2**
YIL076W	SEC28	33	MDYFN	1	**-5.9**
YNL313C		45	METLL	1	**-5.8**
YOL129W	VPS68	58	MEADD	1	**-5.3**
YJL041W	NSP1	42	MNFNT	1	**-5.3**
YOL086W-A		34	MNDDE	1	**-5.1**
YIL088C	AVT7	46	MEATS	1	**-4.6**
YPL262W	FUM1	41	MNSSF	24	**-4.5**
YLR178C	TFS1	54	MNQAI	1	**-4.3**
YFL023W	BUD27	67	MDLLA	1	**-4.0**
YHR060W	VMA22	53	MDTTD	10	**-3.7**
YLR423C	ATG17	30	MNEAD	1	**-3.7**
YGR231C	PHB2	34	MNRSP	1	**-3.6**
YLR430W	SEN1	54	MNSNN	1	**-3.5**
YLR119W	SRN2	36	MDVVP	31	**-3.5**
YNL044W	YIP3	51	MNQLG	1	**-3.5**
YPR021C	AGC1	43	MEQIN	1	**-3.4**
YDL116W	NUP84	48	MELSP	1	**-3.0**
YDR017C	KCS1	39	MDTSH	1	**-2.9**
YJR089W	BIR1	35	MDGQI	1	**-2.9**
YDL188C	PPH22	34	MDMEI	1	**-2.8**
YCR002C	CDC10	70	MDPLS	1	**-2.6**
YDR129C	SAC6	32	MNIVK	1	**-2.5**
YDL128W	VCX1	79	MDATT	1	**-2.5**
YNL092W		50	MDENE	1	**-2.1**
YGL242C		61	MNTEG	1	**-1.9**
YLR056W	ERG3	66	MDLVL	1	**-1.8**
YER012W	PRE1	44	MDIIL	1	**-1.6**
YEL056W	HAT2	47	MENQE	1	**-1.5**
YDR320C-A	DAD4	50	MENPH	1	**-1.2**
YDL141W	BPL1	39	MNVLV	1	**-1.1**
YBR154C	RPB5	32	MDQEN	1	**-0.3**
YDL122W	UBP1	31	MDLFI	1	**-0.2**

Table listing identified N-acetylated peptides displaying the NatB consensus sequence at the N-terminus (MD/ME/MN). Their ^15^N/^14^N ratios (log2) were averaged across biological replicates and show significant down-regulation in the *nat3Δ *strain compared to the WT.

**Figure 2:**
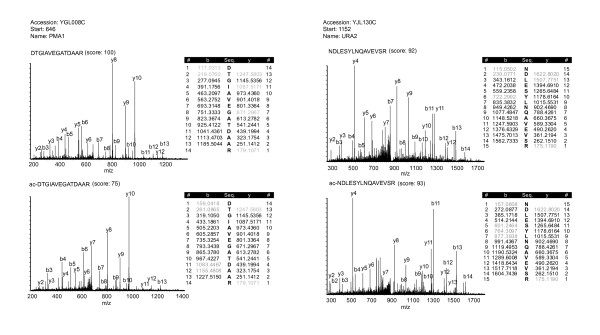
**N-acetylation of protein variants**. Tandem mass spectra of N-acetylated protein variants from Pma1p and Ura2p are displayed. These proteins were found to be N-acetylated on the amino acids N and D as suggested by the 42 Da mass shifts of the b-ion series compared to the same peptides in their non-acetylated forms, which are displayed in the top row. Fragment ions of the y and b series found in the MS/MS spectra are indicated in black in the tables next to the spectra. Missing ions are marked in grey. For each spectrum the start position of the respective peptide is indicated together with the peptide sequence, protein name, accession and MASCOT score.

Notably, our targeted analysis also revealed extensive N-acetylation of peptide N-termini, not originating from the predicted ultimate or penultimate gene-starting position (Additional file 5), as earlier reported to occur also in human cells [25] and *Drosophila *[38]. Figure 2 displays "internally" N-acetylated peptides of Pma1p, a proton pump located in the plasma membrane and of Ura2p a bifunctional enzyme that catalyzes the first two steps of pyrimidine biosynthesis. The MS/MS CID spectra of these peptides, which appear in the acetylated and non N-acetylated form, show a similar fragmentation behavior. Furthermore the 42 Da mass shift of the entire b-ion series clearly indicates the location of the acetyl group at the peptide N-terminus. Utilizing semi-tryptic and semi-Lys-N database search strategies, we identified 250 of such peptides with a minimum MASCOT score of 30 (Additional file 5). Such data provides information to improve protein annotations in databases and offers the ability to study protein processing events on a systemic level. Further analysis of our data intriguingly indicates that N-acetylation can also occur as a genuine post-translational modification instead of co-translational.

**Figure 3:**
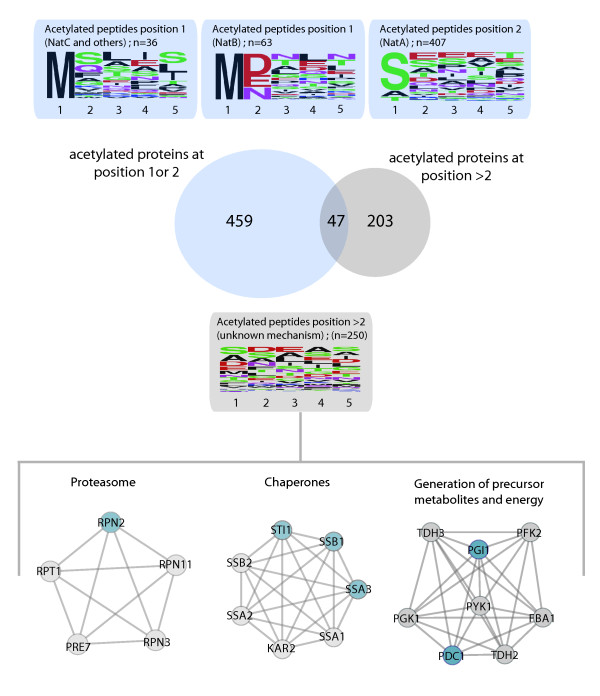
**Profiling N-acetylation in yeast**. A Venn diagram representation of the overlap between identified N-acetylated proteins carrying the N-acetylation on position 1 or 2, and protein variants detected to display N-acetylation on amino acid position 3 or higher. Sequence logos were calculated for peptides acetylated at position 1 and 2 from the predicted gene-start. For position 1, sequences were devided into peptides that matched the NatB consensus sequence and the rest which is most likely acetylated by other N-acetyltransferases such as NatC. Acetylation in position 2 was found to follow the consensus sequence of NatA. Frequency logos are displayed at the top in the blue frames. The frequency logo for proteins N-acetylated on a amino acid higher than 2 (from the predicted gene-start) are indicated below in the grey panel, revealing no particular consensus sequence for this latter category. Network analysis was performed on these latter protein variants and the three main protein clusters are indicated below the frequency logo. These protein variants were found to be preferentially involved in the proteasome, chaperone network and energy metabolism. Proteins detected to be N-acetylated either in position 1 or 2 and additionally at a position higher than 2 are indicated in blue in the protein clusters. All detected protein variants are given in Additional file 5.

Amino acid frequency analysis of the acetylated residues of these over 200 "internal" acetylated N-termini showed that there was no clear consensus sequence, in sharp contrast to proteins that are acetylated at position 1 or 2, i.e. specifically by the N-acetyltransferase complexes NatA or NatB (Figure 3). This might infer the presence of an alternative and more promiscuous N-acetylation mechanism. Strikingly, several proteins such as Cdc19p, Fba1p, Ura2p, and Pgk1p contain several of these "internal" N-acetylated termini. For instance, for Ura2p we detected 5 N-acetylated internal residues apparently at position 602, 684, 1152, 1332 and 1403. Moreover, for some of these proteins the same internal termini could be detected in their non-acetylated form (e.g. Ura2p, Pma1p, and Pgk1p). These findings point to that some of these protein variants seem to be partially acetylated on N-terminal residues like asparagine, proline, leucine, aspartic acid, or isoleucine (Additional file 5), all not the usual targets of the common N-acetyl transferases. Network analysis of these internally cleaved and modified protein variants revealed three main clusters with a prominent representation of the proteasome, the chaperone network of the HSP70 family and energy metabolism (Figure 3). Obviously, many of these proteins are also highly abundant, which may also play a role in the explicit observation of the internally cleaved, and N-acetylated, forms of these proteins. Interestingly, for 35 of those protein variants we also could detect the regular acetylated N-terminus at position 1 or 2. Examples for this are Rpn2p, which is part of the proteasome and Ssa3p, Ssb1p and Sti1p, which belong to the HSP70 chaperone family. It remains to be seen whether this category of internally cleaved and N-acetylated protein variants are generated co-translationally or are cleavage products of proteases, but their appearance cannot be discarded. Of these peptides 33 do either start or are preceded by a methionine, which would indicate an alternative translation start site (Additional file 5). It should be noted that the isotopic ratios of most of these internal termini between WT and *nat3Δ *did not change. Four N-acetylated internal peptides from the proteins Vma22p, Sti1p, Fum1p and Srn2p, however, displayed down-regulation in the *nat3Δ*. Interestingly, those peptides show the N-terminal NatB target sequence (Table 1) indicating that the corresponding genes have most likely alternative translation start codons as indicated by the N-terminal methionine of these peptides. Thus, such genes apparently produce protein variants that are co-translationally modified by the NatB complex.

Next, we shifted our attention to the impact of *nat3Δ *on general protein and protein phosphorylation levels. Protein levels (n = 2580) showed a quite narrow centered distribution with only 2.4% (63 proteins) of quantified proteins displaying a more than 3-fold increase in abundance while only 1.2% (32 proteins) showed down-regulation (Figure 1C). In sharp contrast, protein phosphorylation levels were clearly and significantly increased in the *nat3Δ *strain. 23% (489 phosphorylated peptides) of all quantified phosphorylated peptides displayed a more than 3-fold up-regulation (Figure 1D), whereas only 3.5% (78 phosphorylated peptides) displayed decreased levels. Notably, this increased phosphorylation was clearly evident in both biological replicates, including the isotope label swap.

### Effect of nat3Δ on protein levels

The phenotype of the *nat3Δ *strain is quite complex and the consequences on growth rate suggest that changes in overall protein levels could be expected. To investigate *nat3Δ *downstream effects on the cell we were able to quantify 2580 proteins (excluding quantified proteins outside the 95% confidence interval) and subjected proteins displaying a more that 3-fold change (i.e. less than 100 proteins) to a network and cluster analysis. In contrast to proteins with decreased abundance levels, proteins with increased levels showed interesting associations and localization. Amongst the higher expressed proteins in the *nat3Δ *strain we detected a cluster of nuclear proteins involved in ribosome biogenesis (Nob1p, Cic1p, YNL110C, Nop4p, Nop12p). Interestingly, even though the biogenesis of the ribosome seems to be affected, ribosomal proteins themselves did not display a change in abundance (average ^2^log ratio of ribosomal subunits was 0.02 ± 0.1).

Other proteins with increased expression in the *nat3Δ *strain are involved in cytokinesis and budding such as the kinase Hsl1p, which is involved in septin assembly and linkage of morphogenesis to mitotic entry [39]. Another protein, Chs1p is responsible for the synthesis of the chitin ring involved in bud emergence and cytokinesis [40]. This is particularly intriguing since it is known that the phenotype of the *nat3Δ *strain shows abnormal budding behavior such as multiple buds [13] and coincides with finding up-regulation of proteins like the glucanases Sun4p and Scw10p or the endochitinase Cts1p, which are associated with cell wall separation and therefore morphogenetic events such as budding.

### Effect of nat3Δ on protein phosphorylation

**Figure 4:**
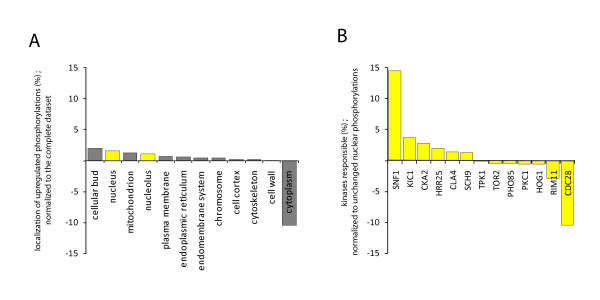
**Localization of up-regulated phosphoproteins and kinases predicted to be responsible for elevated phosphorylation levels**. **Panel (A) **Bar chart indicating localization of proteins displaying significantly increased phosphorylation levels. The % of proteins being localized in the respective categories was calculated for up-regulated proteins and normalized to the localization distribution determined for all detected proteins. The yellow color highlights proteins of the nucleus and nucleolus. **Panel (B) **Bar chart indicating kinases predicted to be responsible for the observed elevated phosphorylation sites. The % of phospho-sites being targeted by the respective kinases was calculated for up regulated sites and normalized to the background of detected unchanged phospho-sites, revealing the predominant role of SNF1 in the observed increased phosphorylation in the *nat3Δ *strain.

The *nat3Δ *strain displays a very clear increase in phosphorylation levels. A localization analysis of proteins that display this increase in phosphorylation levels showed that the main effects seem to take place mainly in the cellular bud (p-value = 0.01) but also in the nucleus (p-value = 0.3) and the mitochondria (p-value of 0.14), while the cytoplasmic compartment is underrepresented (p-value = 0.01) (Figure 4A). To dissect the underlying kinase networks, we used several tools to predict the kinases responsible for the sites displaying increased phosphorylation levels. The results of these predictions are listed in the Additional file 3. To pin-point the prominence of particular kinases we calculated the contribution (in %) of each predicted kinase to elevated phosphorylation sites. This percentage was then normalized by the contributions of the respective kinases to the unchanged nuclear phosphorylation sites. These analyses point out that the serine/threonine kinase Snf1p is most prominently involved in the observed elevated nuclear phosphorylation levels (p-value = 0.004) (Figure 4B). A similar trend for Snf1p could be observed when looking not only at the nuclear subset of elevated phosphorylation sites but at the complete dataset (data not shown) indicating a general increased activity of Snf1p, which can be localized in various cellular compartments [41]. Snf1p influences a large protein network and is, amongst other things, responsible for energy regulation and glucose derepression by transcriptional activation [42], [43].

**Figure 5:**
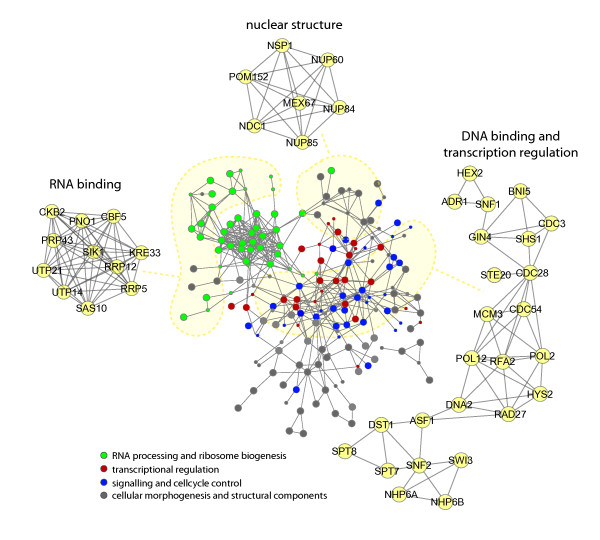
**Functional association of proteins increased in phosphorylation**. Protein networks illustrating associations between up-regulated phosphoproteins. In the middle the total network is depicted, including a rough functional classification of the mapped proteins. Around this central network are depicted the three most prevalent nuclear protein clusters.

In agreement, network analysis illustrated that the effects of the Nat3 deletion affects a large phosphorylation network, stretching to various cellular locations and functions (Figure 5). Alongside structural and scaffold elements such as proteins involved in transport e.g. Hxt3p and Tom6p or protein folding e.g. Ssc1p, elevated phosphorylation levels are also observed for proteins involved in cell cycle control, for example Slt2p, Ms1p or Cdc28p. The main protein clusters extracted from this network analysis consisted of nuclear proteins involved in RNA processing such as the proteins Pno1p, Cbf5p, Sik1p Rrp12p and Utp14p. Other proteins belonging to this cluster play important roles in the biogenesis of ribosomal proteins. Other relevant elevated phosphoprotein clusters were found to be involved in the structural elements of the nucleus such as the nuclear pore complex (e.g. Nsp1, Nup60, Nup84p and Nup85p), and proteins involved in DNA metabolism (e.g. Rad27p, Rfa2p, Dna2p, Pol2p and Pol12p), cell cycle progression (Cdc28p, Cdc54p) and transcriptional regulation (e.g. Spt7p, Spt8p and Snf1p). These results suggest possibly a primarily nuclear localized effect of *nat3Δ *on protein complexes and networks involved in RNA processing (Figure 5).

## Discussion

### The yeast N-acetylome and NatB substrates

Using a comprehensive quantitative proteomics approach enabled us to characterize protein level changes in a *nat3Δ *yeast strain leading to the experimental observation of 756 acetylated protein N-termini, of which 59 (8%) substrates of the NatB complex, expanding the list of NatB substrates significantly. Our data confirmed that NatB has a very high specificity in yeast and exclusively N-acetylates protein sequences starting with MD, ME and MN. Analysis of the yeast genome revealed that 4012 N-terminal protein termini should theoretically be detected using our proteomics approach (our technique is more or less able to measure N-terminal peptides from 5 to 45 amino acids in length). 636 (16%) of the theoretically observable proteins display an N-terminal NatB target sequence (Additional file 6). The discrepancy between the theoretically possible and experimentally detected protein N-termini and NatB targets can be attributed to several sources. First of all, we primarily only enrich N-acetylated protein termini and it has been shown that in yeast only 60-70% of the protein termini are modified in this way. Thus it is very likely that not all proteins that possess the N-terminal NatB target sequence are actually N-acetylated *in-vivo*. Moreover, proteins of very low abundance (copy numbers) may not be detected, even by our targeted approach.

**Figure 6:**
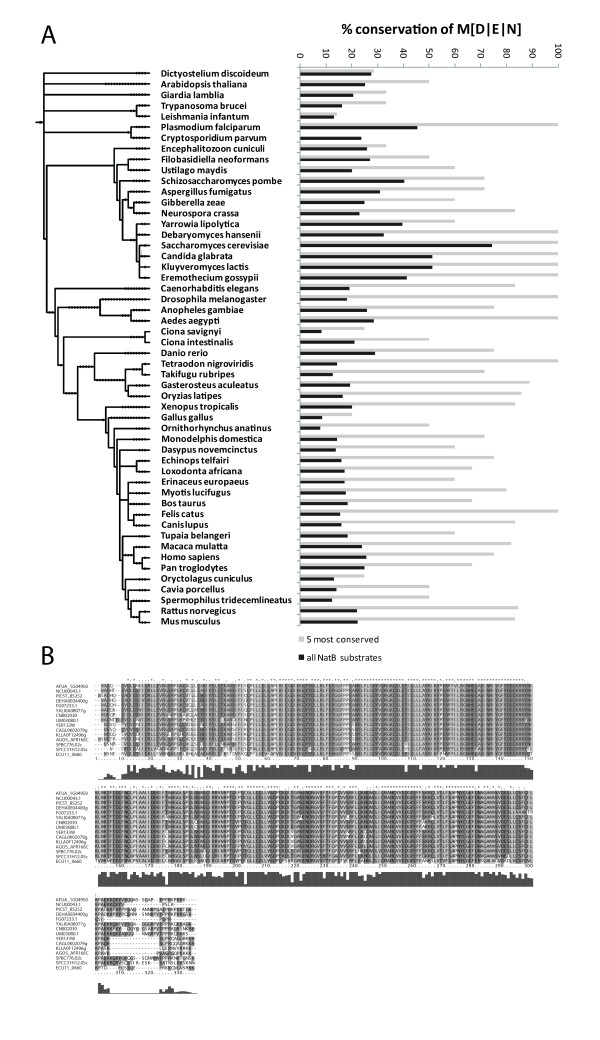
**Conservation of NatB substrates across species**. **Panel A **displays a bar chart indicating the conservation of NatB targets across species. This analysis was performed using either all 59 identified NatB substrates (black bars) or only the 5 most conserved proteins Arp2p, Bos1p, Erg3p, Rpb5p and Rps28ap (gray bars). NatB substrates are only sporadically conserved in the tree of life with the exception of a few, highly conserved, proteins. The phylogenetic relationship between the species included in this survey is indicated on the left. **Panel B **shows an alignment of Glc7p with orthologous protein sequences from different species of the fungal kingdom indicating general high conservation at the full-length protein level. The termini, however, are much less conserved including the part that determines N-acetyltransferase substrate specificity.

The different known N-acetyltransferases have conserved specificities across species and act on a largely identical subset solely determined by the first 1 or 2 N-terminal amino acids [44]. We assessed the conservation in this ultimate N-terminal region of the here detected NatB substrates across several species. Therefore, we extracted orthologous protein sequences from various species and aligned and compared their N-terminal sequences. Surprisingly, the targets of NatB do not show a particular conservation across the phylogenetic tree and only a few highly conserved proteins (Arp2p, Bos1p, Erg3p, Rpb5p, Rps28ap) are apparently showing a consistent N-terminal NatB substrate consensus sequence (Figure 6A), indicating that the N-terminal protection by an acetyl group may not be very tightly associated with a specific N-acetyltransferase. For instance, an alignment of orthologous sequences of the phosphatase Glc7p, which was found to be a NatB target, shows that the protein is in general very well conserved; however, the N- and C-terminal regions display a much lower degree of conservation (Figure 6B), making Glc7p not a NatB substrate in even closely related species. This analysis indicates that caution should be taken when translating phenotypic results from an N-acetyltransferase deletion strain from *S. cerevisiae *to other organisms.

### nat3Δ downstream effects

One of the main reasons for performing this work originates from the fact that the complex phenotype of the *nat3Δ *strain in *S. cerevisiae *cannot be easily explained by just the previously described NatB substrates. In our analysis, we identified several "new" NatB substrates involved in processes impaired in the *nat3Δ *strain. The NatB target Bud27p, for example, is involved in bud site selection and its KO leads to a random budding pattern similar to the budding behavior in the *nat3Δ *[45]. The kinase Hsl1p, which is involved in septin ring formation during cell division [46] was found with elevated levels in the *nat3Δ *and could also be involved in the impaired budding phenotype. However, since the underlying mechanism of Bud27p function is not well characterized, also the impact of its (lack off) N-acetylation status remains elusive.

The reported inability of the *nat3Δ *strain to form functional actin cables is likely due to the loss of the N-acetyl group in actin, but we also found two other NatB substrate proteins functionally associated with actin (Arp1p [47] and Sac6p [48]), that could further contribute to the loss of function. The observed increase in temperature sensibility [49] of the *nat3Δ *strain could be related to the heat shock protein Hsp104p, a NatB target and involved in thermo tolerance and stress response [50]. Further, the defect in mitochondrial inheritance observed when disrupting the NatB complex [51] could be attributed to the loss of the N-acetyl group of Ugo1p, a protein which is located in the mitochondrial outer membrane where it is required for mitochondrial fusion [52].

One of the most intriguing findings in this work is that we detected Glc7p, a serine/threonine phosphatase [53], as a NatB target. This protein is an important regulator and involved in many processes including energy metabolism and G2/M cell cycle progression [54], [55] and interestingly regulates SNF1-mediated phosphorylation, which was observed to be increased significantly in the *nat3Δ*. Considering the slow growth rate displayed by the *nat3Δ *mutant, our data, as well as data from Caesar *et al*. 2006 [13], suggests that defects are not simply caused by the loss of functional actin cables. Instead the interplay of a variety of NatB substrates and further downstream effects may have even larger effects on for instance cell cycle control, cell metabolism and morphology. Especially changes in phosphorylation networks may mediate signals and control cellular functions such as the cell cycle [56], [57]. There is no obvious direct link between the identified NatB substrates and the observed drastic effect of the Nat3 deletion on protein and phosphorylation levels. Analysis of phosphorylation levels in the WT and *nat3Δ *revealed a clear increase of phosphorylation levels in the *nat3Δ *strain. Evaluation of protein networks derived from elevated phosphorylation sites in the *nat3Δ *strain showed that the main affected phosphoprotein clusters could be found in the nucleus of the cell. Furthermore, kinase prediction indicates that the Snf1p kinase is significantly (p-value = 0.004) involved in phosphorylating elevated nuclear (and cytosolic) sites. Our data, however, also shows that protein levels of Snf1p do not change significantly in *nat3Δ*. Snf1p becomes activated during glucose deprivation [58], [59] and gets then localized to the nucleus, where it is involved in controlling transcriptional activators, repressors and RNA polymerase II. As such Snf1p has a strong influence on the regulation of the cellular metabolism [41], leading to the derepression of glucose related genes, inducing adaptation to a nutrient poor environment by e.g. increased glycogen accumulation [60]. As a consequence, proteins such as Hxt7p, which belongs to the hexose transporter family and is normally repressed at high glucose levels [61], will be derepressed. Strikingly, we found Hxt7p to be around 3-fold up regulated in the *nat3Δ *strain. Reg1p, a known regulator of the Glc7p phosphatase, is known to be phosphorylated by Snf1p during glucose limitation and becomes de-phosphorylated by Glc7p after glucose addition.

Snf1p mutations result in the inability of yeast to accumulate glycogen as energy storage, when grown on rich media [60]. In our context, however, we see a hyperactivity of Snf1p which in turn could lead to an activation of glucose repressed genes. The resulting increase in glycogen accumulation is indeed one of the phenotypic characteristics of the *nat3Δ *strain [62]. A likely explanation for this *nat3Δ *effect could be a disruption of the regulatory interaction network between the phosphatase Glc7p, Reg1p and the kinase Snf1p. We clearly show that Glc7p is a NatB substrate, its N-terminus being acetylated in the WT strain. We suggest that the loss of N-acetylation could impair the proper function of this phosphatase in the *nat3Δ *strain. We observe hyper-phosphorylation of Reg1p (Additional file 3) indicating that the interaction and subsequent de-phosphorylation by Glc7p is impaired. This is known to affect the phosphorylation status of the Snf1 kinase [63]. In agreement, we found increased phosphorylation of Snf1 at sites S443 and S487. Both of these residues are localized in the Snf4-interacting domain of Snf1p [64] suggesting that phosphorylation at these residues regulates interaction with Snf4p and hence Snf4p-mediated release of auto-inhibition of the Snf1 kinase [65]. As a result, various targets of the Snf1p kinase could display elevated phosphorylation levels in the *nat3Δ *strain, as observed in our data. Alternatively, there is the possibility that Glc7p acts directly on Snf1p substrates. An impaired Glc7p function in the *nat3Δ *strain could then also have a more direct effect on the phosphorylation levels.

## Conclusions

We applied a system-wide proteomics strategy to identify substrates of the N-terminal acetyltransferase NatB in *Saccharomyces cerevisiae *uncovering 59 proteins lacking N-acetylation in a *nat3Δ *strain. A bioinformatics survey of protein orthologous of these identified substrates in various species showed that the conservation of NatB mediated N-acetylation is infrequent throughout the phylogenetic tree. Further, we present evidence of protein variants with non-annotated N-termini that are also N-acetylated; however their N-terminal sequence doesn't seem to contain conserved motifs in contrast to regular N-termini and may be results of none-co-translational N-acetylation. In addition, we investigated the downstream effects of Nat3 deletion on protein and protein phosphorylation levels to gain insights into the biological role(s) of N-acetylation. We revealed a clear elevation of phosphorylation levels in the *nat3Δ *strain showing, for the first time, an influence of N-acetylation on phosphorylation networks. The kinase Snf1p is apparently a key element responsible for this effect.

## Authors' contributions

AOH carried out all experiments, performed the proteomics analysis and interpretation of the data, and drafted the manuscript. SR assisted in the proteomics analysis and interpretation of the data. WWMPP and HthMT did assist in the growth of the yeast strains and the ^15^N isotope labeling. BvB supported the bioinformatics analyses, including the statistical analysis. AOH, SM, MS and AJRH conceived the study and wrote the paper. MS participated in its design and coordination. All authors read and approved the final manuscript.

## Supplementary Material

Additional file 1 **Table S1. N-acetylation**. displays an inventory of acetylated protein N-termini in *S. cerevisiae*. Click here for file

Additional file 2 **Table S2. Protein levels**. displays 15N/14N isotopic ratios of protein levels comparing WT and *nat3Δ*. Click here for file

Additional file 3 **Table S3. Posphorylated peptides**. displays quantified phosphorylated peptides from the WT and *nat3Δ*. Click here for file

Additional file 4 **Table S4. NatB substrates**. displays an inventory of detected NatB substrates. Click here for file

Additional file 5 **Table S5. Protein variants**. displays an inventory of detected protein variants. Click here for file

Additional file 6 **Table S6. In-silico digestion**. shows detectable N-terminal peptides after *in-silico *digestion using trypsin or Lys-N. Click here for file
